# GENE2D: A NoSQL Integrated Data Repository of Genetic Disorders Data

**DOI:** 10.3390/healthcare8030257

**Published:** 2020-08-06

**Authors:** Halima Samra, Alice Li, Ben Soh

**Affiliations:** 1 Department of Computer Science and Information Technology, La Trobe University, Melbourne, VIC 3086, Australia; B.Soh@latrobe.edu.au; 2 Faculty of Computing and Information Technology, King Abdulaziz University, Jeddah 21589, Saudi Arabia; 3 La Trobe Business School, La Trobe University, Melbourne, VIC 3086, Australia; A.Li@latrobe.edu.au

**Keywords:** integrated data repository, IDR, genetic data, NoSQL document store, MongoDB, Saudi genetic research, clinical data integration, NoSQL-based integration framework, Saudi Arabia

## Abstract

There are few sources from which to obtain clinical and genetic data for use in research in Saudi Arabia. Numerous obstacles led to the difficulty of integrating these data from silos and scattered sources to provide standardized access to large data sets for patients with common health conditions. To this end, we sought to contribute to this area and offer a practical and easy-to-implement solution. In this paper, we aim to design and implement a “not only SQL” (NoSQL) based integration framework to generate an Integrated Data Repository of Genetic Disorders Data (GENE2D) to integrate data from various genetic clinics and research centers in Saudi Arabia and provide an easy-to-use query interface for researchers to conduct their studies on large datasets. The major components involved in the GENE2D architecture consists of the data sources, the integrated data repository (IDR) as a central database, and the application interface. The IDR uses a NoSQL document store via MongoDB (an open source document-oriented database program) as a backend database. The application interface called Query Builder provides multiple services for data retrieval from the database using a custom query to answer simple or complex research questions. The GENE2D system demonstrates its potential to help grow and develop a national genetic disorders database in Saudi Arabia.

## 1. Introduction

The delivery of healthcare in the health sector results in a large amount of patient information that is usually spread throughout an organization’s health information systems. Some are administrative and claim data, and others are clinical data. Reuse of clinical data is essential to deliver quality healthcare, improve healthcare management, reduce healthcare costs, manage population health, and maintain efficient clinical research [1]. In addition, data are a crucial resource for most health and medical research [2]. However, making clinical data available for researchers is a difficult task facing most university teaching hospitals and research centers [3]. This is due to the way the data are captured and used by various health information systems (HISs), which result in data fragmentation and information siloed within different databases and repositories [2]. Furthermore, the presence of clinical data in disparate local storages within each institution in various forms and formats prevents the use of analytical tools to mine through the data at a group-level as well as hindering data integration and sharing among different organizations [4]. The problem of clinical data provision for researchers in medical studies in Saudi Arabia exists at multiple levels. First is the inadequate implementation of the electronic medical record (EMR), a lack of a standard format of data collection, and poor traditional data collection and management methods used for medical research [3,5]. Second is a lack of standards and integration profiles to enable information exchange among healthcare information systems; as the healthcare system in Saudi Arabia is delivered by multiple agencies, governmental and private organizations with different data protection and sharing policies [6]. This has prevented the establishment of a national database for genetic disorders as well as a database for genetic variations reported by diagnostic and research laboratories [7].

## 2. Background

Saudi Arabia is one of several countries that has been affected by inherited diseases due to the high rate of consanguinity which is a powerful factor in shaping the landscape of genetic disorders and accelerating the annotation of recessive Mendelian genes [8]. The kingdom is keen to reduce the occurrence of genetic disorders through premarital and prenatal medical examinations, including genetic screening tests [9]. Genetic testing services are available through government and private labs, mainly the Saudi Diagnostics Laboratory (SDL) which was established by the King Faisal Specialist Hospital and Research Centre (KFSHRC) to support the development of molecular genetic testing and biomedical research in the Kingdom. In addition to other genetic tests which can be conducted through the KFSHRC laboratories, for example, the national program for newborn screening for inborn errors of metabolism, the premarital screening program for Mendelian disorders, the prenatal and the preimplantation genetic for the diagnosis of single-gene disorders [8].

In order to obtain genetic data for the study and develop preventive measures and treatment of genetic diseases in the region, the Saudi government encouraged the establishment of biobanks using biological samples from affected and/or healthy individuals. The establishment of several Saudi biobanks provided researchers access to Saudi biological specimens along with health information which constituted the primary Saudi biobank database, to be used for a better understanding of diseases affecting the Saudi population [10].

The Saudi authorities created an environment for research and training and established centers of excellence in different regions of the Kingdom, such as the Center of Excellence in Genomic Medicine Research (CEGMR) at King Abdulaziz University in Jeddah, which specializes in conducting transitional research in the area of personal medicine [11].

The genetic studies conducted on the Saudi population have contributed significantly and effectively to the global effort of identifying the common mutations of autosomal recessive genetic disorders in particular [12]. Although Saudi researchers have contributed to global efforts in gene databases, there is still no national database of genetic disorders in the Saudi population. Consequently, there has been a lack of large population-based research in the region [10].

Recently, Saudi Arabia interacted with the global initiative, the Human Variome Project (HVP) to collect and curate all human genetic variations affecting human health. Although they accomplished all the foundation work for the creation of a country node of a centralized national database of genomic mutations, limitation in infrastructures, laboratories capabilities, communications and other obstacles delayed the country’s participation in the HVP [13].

Although Saudi Arabia was one of the Arab countries which contributed to the Catalogue of Transmission Genetics in Arabs (CTGA) database for genetic disorders in Arabs by recording locally discovered genetic disorders which were published in scientific reports at the international level [14], there is still a need for a national genetic disease database of locally detected and prevalent cases of genetic diseases in Saudi Arabia, which will effectively contribute to the CTGA database and the larger scientific community.

## 3. Objectives and Contributions

The provision of genetic data for clinical research is a significant area of study. To date, there has been little investigation into a foundation for future national genetic disorders databases. Also, there is a need for a health informatics framework that facilitates diagnostic workflow and aids in decision-making, enabling information reuse in medical research and public health. To this end, we first investigated the challenges Saudi physicians face in our previous work in relation to the real issues hindering clinical research, such as data provision, integration, and sharing among Saudi hospitals [3]. Our study findings highlighted critical obstacles and unveiled the actual process of conducting clinical research in such a difficult environment. Based on these findings, we decided to find a solution to the lack of an efficient system that is able to contribute to the process of managing data, while diagnosing genetic conditions in Saudi genetic clinics and research centers. As a result, the novel G3DMS (genetic disorders diagnosis data management system) was designed with a view to being applicable in any genetic clinic or research center [15]. Next, in this paper, we further aim to find a solution to solve the issue of data integration. The proposed solution aims to provide large datasets for clinical research from multiple sources of genetic diagnostic data systems. To achieve this aim, we incorporate an integration framework into the G3DMS [15] using an integrated data repository based on the “not only Structured Query Language (SQL)” (NoSQL) database. The design and implementation of the NoSQL-based integrated data repository (IDR) of genetic disorders data (GENE2D) is demonstrated in this paper.

More specifically, our contributions are as follows:

- new mapping rules for extraction based on specified criteria.
- new mapping rules for transforming data from an SQL source format to a BSON (Binary JSON (JavaScript Object Notation)) destination format.
- new mapping rules for loading and indexing data to the destination.
- innovative method for the design of the integrated data repository based on the NoSQL document-oriented database (MongoDB)
- novel Query Builder methods to serve (simple, complex queries) in genetic-related studies and public health research.
- query-performance evaluation of GENE2D.

## 4. Theoretical Foundations

Health information systems in any health organization comprise different software solutions for data collection, storage, and management. Data resides in different locations within the same organization in various formats and structures, and this makes data manipulation a complicated task. A lack of medical record integration among an organization’s physicians or across institutions creates great difficulties in data analysis and medical research studies [16]. In addition, the current patient clinical care databases are often inadequate to assess health interventions because data are often missing or incorrect, and it is difficult to link patients’ demographics and clinical care procedures [17]. Therefore, healthcare institutions need fundamental changes to the infrastructure and mechanisms for data collection, storage and exchange. In order for this data to be suitable for use in research, they must have the ability to be integrated to create a comprehensive view and provide meaningful insights for patients and researchers. But integrating databases with different specifications for data models, database schemas, the queries they support, and the terminologies they use, is a very challenging task. Data sharing through databases is a more common practice in clinical research as data collected at multiple sites are integrated with some disease-oriented database systems, since one location may not be able to collect sufficient data for analysis. Clinical institutions may also have limitations in terms of research interests so a common database can make the collected data available to researchers in a variety of locations [18]. The process of data integration needs to bridge the scientific methods and specifications used in a database. The overall goal of data integration for the clinical research community is to be able to answer questions about aggregated data, which can be very difficult if each individual data source must be accessed separately or sequentially.

### 4.1. Integration Approaches 

Data integration is a prerequisite for obtaining a unified view of clinical and genetic data from multiple operational data stores. The integration of data stored in heterogeneous Database Management System (DBMS) can be achieved by aggregating across data sources using an intermediate solution to retrieve data or combine copies of data in a centralized system from multiple sources. This is to provide a unified view using various techniques for data extraction. The process of data integration involves extracting, transforming, and cleansing the data before aggregation. Several approaches have been employed to integrate data from different sources using warehousing approaches, also known as physical integration (combining a copy of the data in a new repository for further analysis) or using mediation-based approaches known as logical integration (applying conceptual schemas to bridge the representational heterogeneity of the databases, thus providing queries with the ability to collect and integrate data from distributed sources) [19].

#### 4.1.1. Logical Integration 

Logical data integration, also called virtual integration, is based on data which are distributed in their original locations. The mediator software, which resides at a central location and uses a specific query protocol, is the point of communication between the disrupted hosts and users and mediates their request for data. The global schema is used to validate the user query before the mediator uses the mapping information to identify the location of the desired elements for the requested query. The global query needs to be translated into the local DBMS query language of the distributed sources by the mediator. Logical integration approaches require persuasive communication and infrastructure capabilities among health organizations [19]. Currently, this approach does not achieve our objectives of establishing an anonymized national database of genetic diseases data. This is because the limitations in Saudi infrastructures and the adopted technologies do not provide an appropriate environment for logical integration which requires highly advanced information and communication technology [20]. 

#### 4.1.2. Physical Integration

This approach depends on the concept of copying the original data, which is restructured and transferred from one or more sources depending on the scope, purpose, and size of the data. The combined data is stored and managed by these new systems instead of the original source and is categorized into one queryable repository [19]. Enterprise data warehouses (EDWs) or integrated clinical data repositories (IDRs) constitute the major source of the data required for clinical and translational research and they are designed to integrate various aspects of patient care data [21]. To integrate medical data, the design of IDRs should consider several critical issues such as identity management, the protection of confidentiality, convenient and flexible queries, and semantic and ontology definitions at the schema level for data coming from different data sources with different naming and structural conflicts [22]. Data organization and representation in IDR is very important for analysis, as IDR accommodates data from various sources that may require a continuing extension which should be allowed without any major changes in the original schema. This will allow easy maintenance and data analysis [23]. However, in the warehouse approach, integrating heterogeneous data with various formats and structures is a difficult task requiring a global schema to handle data requirements from distributed and extremely complex data structures [24,25]. Modeling IDR with conventional data warehouse (DW) involves specific infrastructure and setups for extract, transform, and load (ETL) procedures and online analytical processing (OLAP) processes as well as reporting tools; all these components are difficult to build and require a lot of organizational resources for implementation and training purposes [25,26]. Although the majority of warehouses are implemented using relational technologies [23], high consistency and availability, their performance decreases with data growth and they face scalability constraints as they are impossible to measure horizontally, and their vertical growth is limited [27]. 

### 4.2. NoSQL Storage Solution

A NoSQL database refers to a non-relational or “not only SQL” database, and is a large distributed open-source system providing a more flexible schema-less data model, which can efficiently store unstructured and semi-structured data [28]. It offers an easier and cost-effective approach to database scaling for a rapidly growing data repository, where horizontal scalability is a feature of NoSQL databases [29]. For example, document-oriented databases are the most popular among the NoSQL families, where the data are stored in collections that are a group of documents, and each document comprises key-value pairs. Document stores are characterized by their schema-less structure which is useful for data with high complexity such as medical records; examples of this type are MongoDB and CouchDB [30].

Although there is a potential for the NoSQL integrative capability and opportunities offered by NoSQL databases and database management systems [31], few studies adopted such a technology in practice in the healthcare domain. Ercan and Lane [32] showed that the requirements of the application can determine the usability of NoSQL databases. Therefore, they suggested that the use of NoSQL databases has great potential for better electronic health record (EHR) applications in terms of scalability, elasticity, and high availability [32]. Feng et al. used a NoSQL database to store integrated data files provided by IDR from the patients’ EHR, so they could apply analytical tools for surgery risk assessment analysis [33]. Also, Tao et al. used the document-oriented NoSQL MongoDB as a backend database for their query framework for data integration to allow researchers better access and to explore large-scale integrated clinical data repositories [21]. Two NoSQL-based patient cohort query systems are used in comparison to a SQL-based system to evaluate their performance in supporting high-dimensional and heterogeneous data sources. The NoSQL databases exceeded the maximum limits for the number of table columns in traditional relational databases and successfully integrated eight datasets into NoSQL databases [34]. Driven by NoSQL document database features such as being opensource and cost-effective, with a flexible schema model, indexing capabilities, and scalability for future expansion, we decided to be among the first to use the NoSQL document store as a back-end for an IDR for genetic data, to the best of our knowledge.

## 5. Method

The physical integration approach is adopted to achieve the intended IDR for GENE2D. The design of the IDR incorporates the ETL process, which will take place at the source system in our case (G3DMS [15]), which has been developed and implemented in a Saudi genetic clinic. We developed our own customized methods for mapping to perform the ETL process. The NoSQL document database is used instead of the traditional relational model. Therefore, our design benefits from the flexible schema model for a complex data structure as document formats are self-describing, and a collection may include documents with different forms [35]. The GENE2D will be accessed via a front-end query interface called a Query Builder, explicitly designed for research purposes and answering ad hoc research questions.

## 6. System Architecture

**Figure 1:**
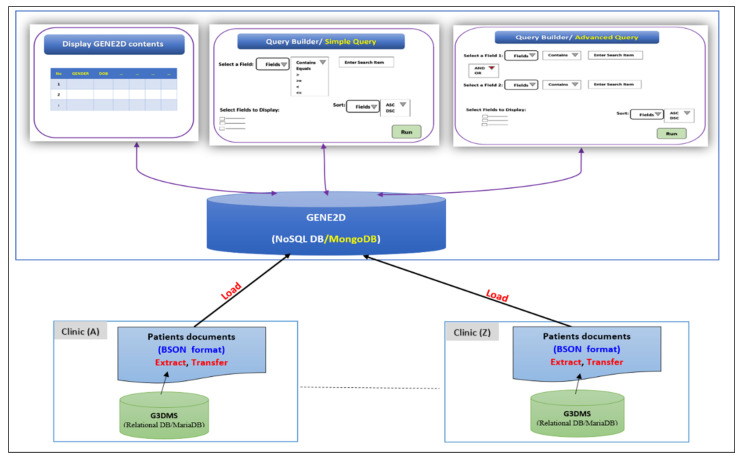
The NoSQL Integrated Data Repository of Genetic Disorders Data (GENE2D) architecture.

The NoSQL integration framework combines data on genetic diseases from multiple sources into the GENE2D system. The major components involved in the design architecture are as shown in Figure 1 are:

- Integration sources: G3DMS contains genetic disorder data stored in relational tables on a MariaDB server, and is the source from which genetic data are extracted, converted and uploaded to the integrated data repository, GENE2D.
- The NoSQL document store, MongoDB, which uses a collection of documents to store the data as BSON (Binary JSON (JavaScript Object Notation)) will be the centralized repository for the integrated data.
- The Query Builder interface that provides multiple pages for data retrieval in selective display forms according to the query type selected; it allows researchers to retrieve contents from the database using a custom query to answer simple or complex research questions.

## 7. Design Methodology

The design methodology of the GENE2D system aims to achieve the final design solution in three fundamental design stages:

- Data acquisition process
- Data modelling
- System implementation and testing

The design process involves automated data extraction transformation and load (ETL) at the source using customized methods. First, the data extraction step results from reading data from the source (G3DMS) [15]) using PHP methods for MySQL. Then a physical schema based on the data structure model is developed. Next, the constructed schema for the target system (NoSQL document database) is used as a model for data transformation using MongoDB/PHP-based methods on the extracted data. After this, patients’ data are loaded into individual documents to reside on the patients collection within the GENE2D system. Last, the GENE2D application interface is implemented, and a validation test is performed.

### 7.1. The Data Acquisition Process

The clinical and genetic data of patients with genetic disorders is available in individual clinics that are collected and managed by the system we developed and implemented, namely the G3DMS [15]. The integration of data from these systems requires a centralized repository (IDR) to accommodate all data coming from multiple G3DMSs. Therefore, when acquiring data from different DBMS with various storage structures, the data cannot be directly migrated unless it goes through different processes to be cleaned, organized, and converted to other formats and types to adhere to the destination structure. Typically, the integration process includes extracting, transforming, and uploading processes, which provide methods for transferring data from the source to the data warehouse database. ETL operations are carried out in a staging area between the source and destination system where data integrity problems are managed and verified before loading. In our design method, the ETL operations will be all executed at the source system automatically using customized methods from both world SQL and NoSQL databases. The extracted and converted data is sent from the G3DMS to the GENE2D system with a simple click of the Export button. The data acquisition process starts with extracting data from the G3DMS, which is cleaned and prepared for transformation to the intended format before uploading to the MongoDB-based GENE2D system.

#### 7.1.1. Data Extraction

The G3DMS contents range from demographic data (medical record number (MRN)), genetic number, name, gender, date of birth (DoB), nationality, address, and contact number), clinical characteristics (phenotypes), family history information (inheritance pattern, consanguinity, mother’s age, DNA availability, pedigree chart, diagnosis data such as samples, consents, tests, results, photos, research papers, reports, conditions, diagnosis status, treatment plans (plan type, description, date), in addition to physicians’ details and contacts [15]. We have imposed several criteria to help with refining data selection and extraction from the source.

#### 7.1.2. Extraction Criteria

- *Patient-oriented*: the integration framework aims to collect patients’ data and create the GENE2D system; therefore, it is a patient-centred database, especially for patients with genetic disorders. Thus, all data extracted should be related directly to the patient, so any other data, such as physician’s information should not be included.
- *Research requirements*: only data that is useful for research related to genetic disorders should be included. Unnecessary data, such as patient treatment plans and contact details, will not be considered. Also, only cases of “confirmed” diagnoses of genetic conditions should be transferred. Therefore, cases with “provisional” diagnoses will be postponed until the diagnosis status changes to “confirmed”. Furthermore, any incomplete patient data which misses important information will not be extracted for data quality purposes.
- *HIPAA compliant*: the GENE2D system contents will be available online to researchers, so they must adhere to specific HIPAA rules and regulations related to the use of identifiable personal health information (PHI) [36]. Since the dataset should not include an identifier which can be used to link back to the patient, any information which may lead to the identity of a patient will not be moved from the G3DMS. These include names, photos, samples, and family pedigree charts or any documents containing patient identification information. However, de-identification can be achieved by masking or encoding a unique key such as the MRN to solve the problem of updating the data in the GENE2D system while preventing duplicate entry.

#### 7.1.3. Extraction Method

The G3DMS uses the Relational Database Management System (RDBMS) MariaDB server as a back-end database for storing data related to patients with genetic conditions. The extraction process is done by applying the following steps. First, a connection to the source G3DMS database is created using PHP- function MySQLi_Connect. Next, the database is navigated using the primary key MRN and making the appropriate linkage to access all data distributed in various tables. Then, each criterion is applied as a condition to the query in the related table to extract only the required fields, for example, the diagnosis status is confirmed. Similarly, some required patient data are retrieved using joints between tables such as patient’s phenotypes/clinical characteristics, and tests and results. Then, the query results are retrieved and accessed to obtain each value and store it in an array of its type. Finally, all the resulting arrays are merged in one single array after checking empty values to ensure that incomplete data is excluded. Now all patient data stored in one array is ready to be converted to the specified format and data type before passing it to the writing method in MongoDB.

#### 7.1.4. Extracted Data

Table 1 lists the data that meet the extraction criteria and is eligible to be copied from the G3DMS to the GENE2D system. The list includes field names, data types/role in terms of their relationship with the primary entity or object “Patient”. All data fields will be moved without any changes except the primary key, the MRN, which has to go through encoding for de-identification purposes and the hashing function is used to produce a new unique ID for each patient in the organization.

### 7.2. Data Modelling

Document-oriented data models deal with documents, so the storage structure is based on the primary unit, collections (tables as in the relational model (RM)) made of individual documents (tuples as in the RM). Documents, in general, contain all the information about the entity collectively, where each document stores a JSON object in the form of key-value pairs, although the document store does not require data modelling where there is no schema definition obligation. Our design methodology supports the data modelling process to generate a schema to define our plan for data organization for better query performance and a quicker response time. Therefore, defining the schema as a proactive step before transferring the data will assist the semantic transformation and increase the overall performance of the application.

Modelling in document databases is as essential as in relational modelling; there is still a schema, but it is not enforced as in relational modelling, although the design of the schema or the development of the data model for a document-oriented database does not follow a standard fixed method such as the relational modelling approach (conceptual, logical, physical) models. A useful data model will result in a robust schema that optimizes query performance while preserving time and storage efficiency. Also, balancing application requirements, database engine performance characteristics and data retrieval patterns are a significant challenge for data modelling. Therefore, application usage must be considered, such as queries and updates, as well as the data structure and organization within the database. Consequently, the purpose of the data modelling process is to produce a useful data model that supports the workflow in the application of the GENE2D system, which aims at retrieving and answering any research question related to patients with genetic diseases. Therefore, the focus is on the read performance being fast and efficient using dynamic querying options.

#### 7.2.1. Schema Plan

The most critical step when developing the data model is the decision on the way entities (objects) should reside in the database. How data is accessed and queried will determine how the data is stored, so data retrieved together should be stored collectively. Since the priority is to increase query performance during the read and update operations, our schema design plan considers minimizing the need for the database to make any joins and reduces Input/Output operations. Hence, the design decision of a patient-oriented database for the GENE2D system is to store all the data sets in one single collection which will encapsulate all the patients’ documents; each patient record will be stored as an independent document. Next, the way the patient’s data is organized inside the document (the physical model) is determined by the data modelling method adopted to develop the physical schema. We follow two design guidelines for schema development. First, the data dependencies and relationships between the attributes and entities of the extracted data from the G3DMS are analyzed, as listed in Table 1. Second, the application-specific access patterns for the GENE2D system are identified, i.e., the query patterns to be supported.

##### Data Dependencies and Relationships

The patient-oriented design focus on the GENE2D system mandates that all attributes and entities in the database are directly related to the patient entity. The patient’s proprietary attributes, such as MRN, gender, DoB, nationality, city, consanguinity, inheritance pattern, and mother’s age, are totally dependent on the patient entity with a relationship of one-to-one. Entities such as conditions, phenotypes, and tests and results, also show dependency on the patient’s entity but with a different relationship of one-to-many.

##### Query Patterns

**Figure 2:**
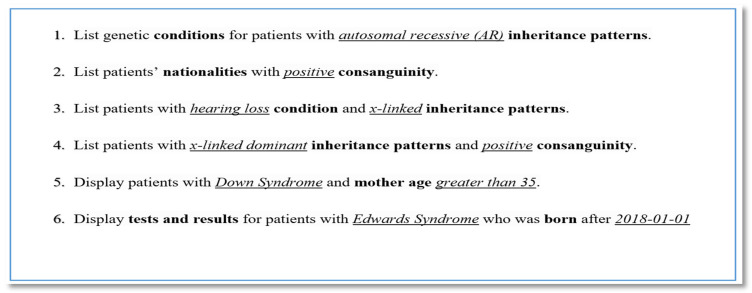
Query patterns represented in use cases.

The purpose of the GENE2D system is to apply it to research studies, specifically to help answer research questions related to diagnostic information for Saudi patients with genetic disorders. Defining the type of queries required by research studies in the area of genetic diseases, to answer “what question to expect?” will determine the data access patterns and the application workflows. Also, it will help in the identification of primary keys, indexes, denormalized attributes and their organization within the document. Figure 2 displays a sample of use cases for questions that can be expected to conduct research in the field related to the diagnostic information on patients with genetic conditions in the Kingdom of Saudi Arabia. The central entity in the database appears to be the “patient” object and its proprietary attributes that are repeatedly present in all queries. Therefore, patient _id is considered as the primary key, and all its attributes need to be denormalized and considered for possible indexing. Also, entities that have shown dependence on the “patient” entity, such as conditions, phenotypes, and tests and results, are subject for indexing too.

#### 7.2.2. Schema Design

Dependencies, relationships between entities and attributes, and query patterns are the key elements in the design of the physical schema. The design focuses on document structure and the representation of data relationships. The results of a discussion of the schema plan and the design guidelines support the choice to store all patient-related data in one collection.

##### Database Structure

**Figure 3:**
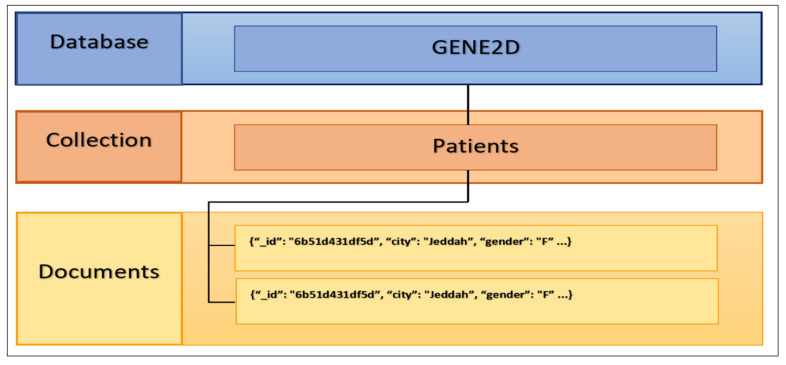
The GENE2D database structure.

Figure 3 presents the general database structure for the GENE2D system in MongoDB, namely the database container “GENE2D”, the collection “Patients”, and the documents where each document denotes patient information represented by the structure of a JSON object. Each patient document or JSON object contains many fields such as _id, gender, and dob. The decision is to aggregate all data needed to process any query in one place so that multiple queries will access the same data in a different combination.

##### Embedded Data Model

**Figure 4:**
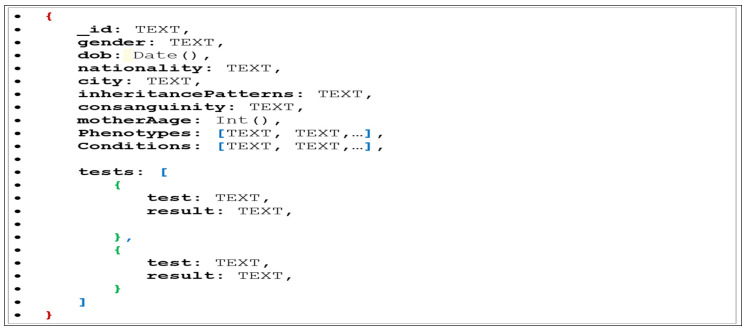
The schema design for the patient document.

The aim is to store the data in a query-friendly structure to facilitate query operations. Therefore, the denormalized or embedded data model to represent the data structure and constitute the physical schema will enable the related data to be stored in a single document which eliminates the need for joins. Embedding refers to the insertion of an individual document within another related element (document) which is frequently used together, i.e., nesting one document into another. First, some of the extracted patients’ properties such as _id, gender, dob, nationality, city, inheritancePatterns, consanguinity, and motherAge, will be included in the “patient” object as flat list attributes(fields), for example {“_id”: “6b51d431df5d”, “city”: “Jeddah”, “gender”: “F”…}. Entities are such as phenotypes and conditions, of (one to many) relationships, the size of the list is limited. Therefore, the decision is to embed both entities as arrays in the “patient” object, for example, {“phenotypes”: [“ph1”, “ph2”,…],…}. Regarding the test entity which consists of a list of {test, result}, there is an option to embed test data as an array of objects inside the patient document or to reference the “test_id” inside the patient document and store the test data in a separate document. We embed and reduce the joins because the tests are bounded with limited growth in size, for example, { “tests”: [ { “test1”, “result2”}, {test2,result2},…], …}. Figure 4 shows the general design of the physical schema, which represents the patient’s document as a JSON object. The overall decision to embed corresponds with the purpose of the application function as a query builder interface.

#### 7.2.3. Transform

Moving data from RDBMS to a NoSQL document store such as MongoDB requires a carefully planned transformation process based on the data structure on both the source and target databases. The schema design is the key for database construction to meet the requirements of the GENE2D application workflow. The transformation process depends on the schema design presented in Figure 4, which is used as a guide for mapping and transforming the extracted data from the source G3DMS to be loaded and stored in the MongoDB destination. Customized methods are implemented to map the extracted data to fall into the schema design structure according to our defined mapping guidelines.

##### Mapping Guidelines

Setting guidelines for data transformation allows us to define and restructure the extracted data using customized operations, for example, for data cleaning and conversion of units such as date and time before loading into the target MongoDB-based GENE2D system.

##### Support Aggregation and a Void Join Operation

Adopting the embedding mechanism will allow all patient data to be accessed together. Therefore, using an array to concatenate all patient data to be mapped in a single operation to a JSON object adheres to the embedding model.

##### Field Naming

The field names in the G3DMS source are consistent for use in research, so expressions are used to name the fields so they can be identified by researchers. Therefore, most field names are mapped directly without changes, whereas slight modifications were made to others, so they are more specific but have the same meaning as (clinical_description => phenotypes), and so on.

##### Multi-Valued Fields

In some cases, a patient may have a list of phenotypes, conditions, and tests. These multi-valued fields need to be stored in a way that they can be retrieved correctly for the individual patient as well as for the cohort. While reading data from the related joint tables of a specific patient using the primary key MRN, we use a multidimensional associative array to store the field name in the key such as array[phenotypes] and a numeric array for storing the values, for example:

Array ([phenotypes] => Array ([0] => pheno1 [1] => pheno2 [2] => pheno3)).

For the tests array, which includes sets of {test, result}, the multidimensional associative array will look like this:

Array ([tests] => Array ([0] => Array ([test] => test1 [result] => result1) [1] => Array ([test] => test2 [result] => result2))).

Therefore, when mapping to the MongoDB document, the array[key] of the associative array [phenotypes] will be used as the field name for the array in the document and the same for the conditions and test arrays.

##### Transforming Methods

*De-identification*

The patient identifier field, the MRN, is the most critical value and so it needs to be treated differently, as it is the conventional method of identifying a patient in Saudi hospitals and clinics. Therefore, our selection of this field to be encoded automatically before sending the data to the GENE2D system is very significant to preserve patients’ confidentiality and prevent duplication of data during the update processes. Our design uses a one-way hash algorithm on the MRN field to anonymize the patient’s data while allowing researchers to update a specific record with a modification. We implemented the standard hash algorithm to transform the MRN string into another string “_id” in a way that no other operation cannot retrieve the original MRN. The “_id” will be assigned as the primary key that uniquely identifies the document.

*Indexing*

When uploading data to MongoDB, each document is indexed automatically using the unique document “_id” as a default primary key set by MongoDB/ObjectId class. But in our case, we assigned the masked MRN as the document primary key. So, the “_id” is set as the index for the document. Indexing on all fields is also valuable in that it makes the reading process more manageable, the query operation much faster and obtains more precise results by reaching all the data inside the arrays and object arrays. One of the significant advantages of MongoDB compared to other document stores is that it allows any field in the document to be set as an index. Therefore, we use the indexing function createIndex([‘$**’ => 1]) to create a wildcard index on all the fields and subfields in a document.

*Convert Fields Types*

Almost all the fields with string type are transformed without any changes, but fields like date type “dob” and integer types “motherAge” must be validated and converted to the appropriate MongoDB format to be valid for a query of its type. For the integer field, a simple PHP (int) function is used to force the type before loading. The “dob” date string in the source database must be converted to conform to the MongoDB format which uses the BSON date format. So, to convert the date, first, we use the PHP function strtotime() to map the date string (y-m-d) into a Unix timestamp. Then, the resulting timestamp is passed to MongoDB to be converted and stored as a BSON date.

#### 7.2.4. Load

##### Loading Methods

The stage of loading the transformed data is preceded by making a connection to a MongoDB server. In our design, we use PHP functions to read from the source in a MariaDB server and write to the MongoDB server using MongoDB classes from the MongoDB library. We use array of objects to store all extracted and transformed data using PHP functions, then a MongoDB function for bulk writing is used to to store the patient’s array contents as a JSON object (document). We use the update operation with the “upsert” option instead of using the regular insert method to allow multiple updates to the same patient document without having the problem of duplicate document error due to “_id” existence in the collection. The insert method works well the first time; however, it fails to write to the collection if there is duplicate “_id” for an existing document. This method can be used to load patients’ data for the first time as well as altering existing patient documents with new data. Executing this method will create a new database if it does not exist, so the database “GENE2D” and the collection “Patients” are created for the first time; otherwise the existing collection is updated with the new entry using the update operation.

### 7.3. System Implementation and Testing

#### 7.3.1. Application Interface

We developed our webpages for the system user interface in the XAMPP platform using the Apache server to run and test the web interface before publishing the GENE2D system on the Cloud.

##### Update the G3DMS Application Interface

**Figure 5:**
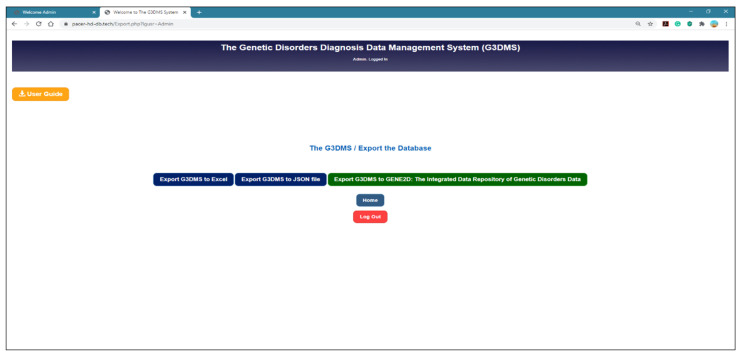
The Export page from the G3DMS to the GENE2D system.

The three main steps in the integration process, data extraction, conversion and load, are combined into one operation at the source G3DMS. To implement the process, we included a button to export the data from the G3DMS to the GENE2D system in the G3DMS application interface, as shown in Figure 5. Furthermore, a PHP file called “export_to_MongoDB.php” was developed in the application files of the G3DMS to read data from the MySQL database tables and then it is converted using classes from the MongoDB Library for PHP.

##### GENE2D Application Interface Development

The primary goal of the system is to provide an easy-to-use user interface taking into consideration novices who may be professionals in their fields but who do not have to worry about learning the query language to conduct their search. Therefore, the user interface design focuses on connecting the system to a user action using clear messages, as well as looking at the simplicity of the user interface and visual aspects such as clarity and color. Since the system will be used by researchers from across the Kingdom of Saudi Arabia, officials in the sector will determine the validity of the entry and the use of the system, but as a first step, access will be granted to users of the G3DMS. The application consists of several webpages for Login, Reset Password, Query Builder Home, Display Database Content, Simple Query Builder, and the Advanced Query Builder.

*Authentication Pages*

Since the system will be accessed by an authorized researcher using the same username and password for the G3DMS, the first page of the application is a login authentication page that verifies the user’s username and password before they are allowed to proceed to the system. The username and password used for the G3DMS authentication are the same. All users’ login data are transferred along with the patient data to be stored in a separate collection called “login” within the GENE2D database. Also, the system allows the user to change their password in the GENE2D system through the Reset Password page, which updates the user information in the “login” collection with the new password.

*The Query Builder Home Page*

This page allows the user to select one of three options to navigate the database, the Display Database Content page to display the entire database, the Simple Query Builder page which relies on one field of the document for answering questions, and the Advanced Query Builder page for querying two fields using a logical operator (and, or) and allowing the result to be sorted (ASC or DESC) by any field.

*Display Database Content Page*

**Figure 6:**
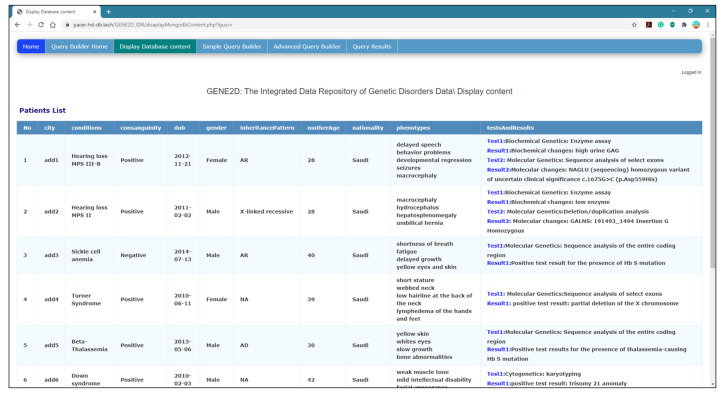
Display of the database content page.

This page creates a new connection to the MongoDB server and reaches the “GENE2D” database and the “Patients” collection. As shown in Figure 6, the resulting data are presented using the HTML table format to display the output in a tabular form.

*Simple Query Builder Page*

**Figure 7:**
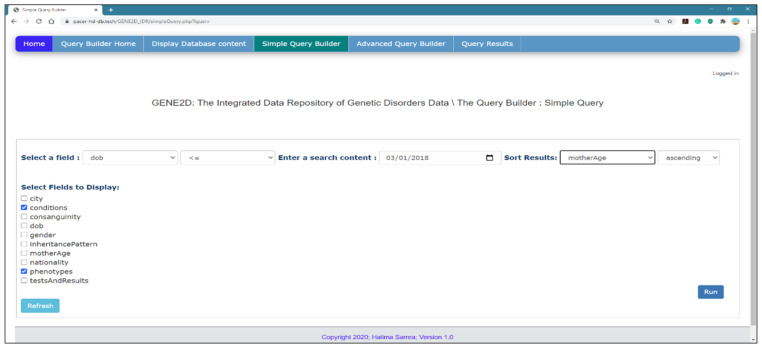
The Simple Query Builder page.

This page allows the user to select a field from the document to search, specify the search criteria, enter search contents, select a field to sort the results and tick all the extra fields of information to display beside the essential selection, as presented in Figure 7.

*Select a field to query*: select an option from the drop-down menu, which displays all fields in the documents. This menu changes dynamically according to the existing fields in the documents. This provides flexibility and guarantees data availability. The user selection from this menu affects the option that appears in the next menu of the search criteria. For example, if the user chooses to query on the field “conditions”, then the adjacent menu will display options (contains and equals), and the text box will allow the user to type a search item. But for the option “consanguinity”, the next menu will change to show options (positive or negative). If “dob” is selected as the search criteria, it will allow a list that matches with the data type, therefore the menu of operators (equals, >,>=,<,<=) appears accordingly; also, a date type insertion box surfaces to allow the selection from the date calendar. Similarly, for the numeric “motherAge” selection, the same search operator shows with an input box for the numeric. When selecting the option of tests and results, a next menu pops up with two options (test or result) and a fixed search criterion of (contains) and a text box to enter the search phrase. We used jQuery functions to control the appearance of menus in the page using hide( ) and show( ) methods according to the user selections. Also, the page is supported by a “Refresh” button to reload the page as well as messages to verify the input fields when the user clicks the “Run” button.

*Sort results*: the same list of current fields appear to select one to sort the query result accordingly with an option to specify the sort order (ascending or descending) that will apply to all data types.

*Fields to display*: Figure 7 shows a list of all field names from the patient document which are displayed next to checkboxes from which the user can choose. Selecting from this list enables the display of additional information as needed to support the research question. This increases the accuracy of the query results and displays the answer from multiple dimensions and perspectives. A verification message appears to remind the user to select at least one field to display if the selection is not made when the query is submitted.

*Simple Query Results Page*

**Figure 8:**
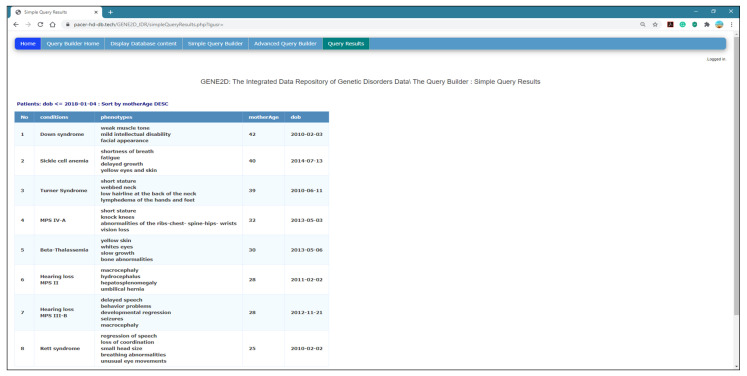
The Simple Query Results page.

This page is called as a response to the submit button “Run” in the Simple Query Builder page. All selections are posted to be managed individually using PHP to retrieve the results from MongoDB and display the query result as output in the HTML table format. The results are organized in a table format with a dynamic table header that reads from the selected fields and user input. Some query results, such as the date, must be set to a human-readable format; thus, the MongoDB to DateTime( ) function is used for this purpose, and then the format function format (‘Y-m-d’) is used to display the date. Figure 8 shows the results of the research question presented in Figure 7: (display conditions and phenotypes for patients who were born in 03/01/2018 and after and sort the results for mother age in descending order).

*Advanced Query Builder Page*

**Figure 9:**
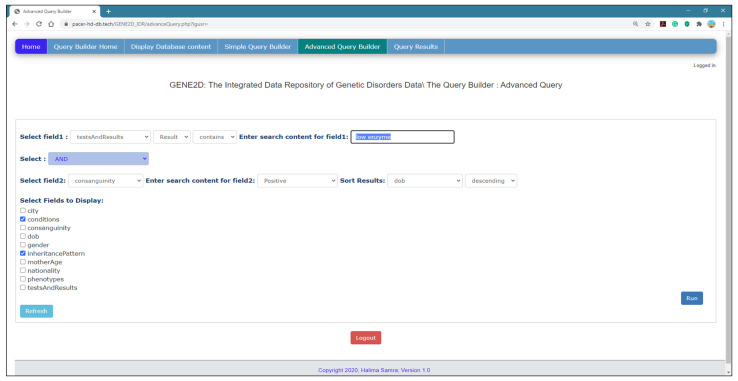
The Advanced Query Builder page.

Figure 9 shows the advanced query page with options for a complex query, and it may include two fields for direct participation for each field in a separate query with the option to link the two queries with the logical operator (and/or) to match the result. Furthermore, similar to the simple query, any field can be used to sort the results as well as display additional information. Also, the results are presented in the form of a table.

*Select fields to query*: this page provides two drop-down menus to select a field to participate in two queries and an options list to choose search criteria and input search elements for each query. Note that the options fields are independent as the user can query in the first list of fields or the second list separately or use both lists.

*Logical operator*: there is an additional list of options to select a logical operator (and, or) to match the results from both queries.

*Sort results*: it allows the results to be sorted according to one of the fields marked in either ascending or descending order.

*Fields to display*: additional information can be provided with the query result by selecting more fields to show from the list as depicted in Figure 9.

*Advanced Query Results Page*

The response on this page takes different paths to perform operations, depending on the options specified in the Advanced Query Builder page. First, if the user decides to use only one field, then the page directs the selection to its specific function to execute the query as it did on the Simple Query Results page if both fields are selected and a logical operator, as well as the other related option for both fields. Then the code directs the selection and entry which is posted from the previous page to be executed depending on the search criteria and the type of fields selected as explained in detail in the Simple Query Results page.

**Figure 10:**
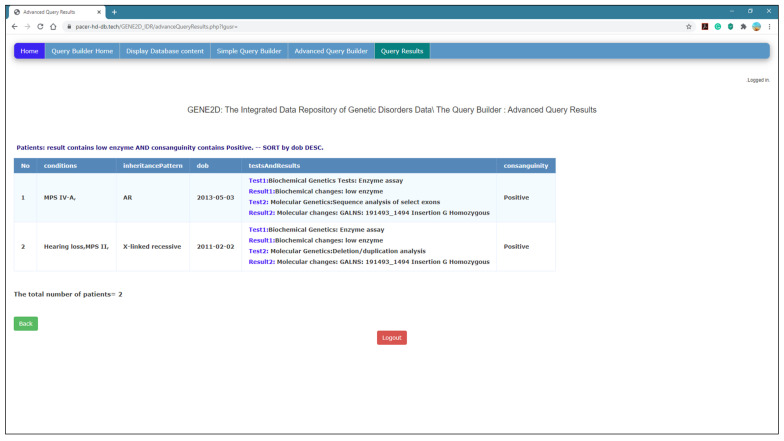
The Advanced Query Results page.

Figure 9 presents the research question: (display the conditions and inheritance patterns for patients with positive consanguinity, and their test results contain “Karyotype”). Figure 10 shows the Advanced Query Results page that displays the results of a complex query presented in Figure 9. The results are organized in a table format with the count of the number of documents retrieved.

#### 7.3.2. Validation Test

##### Query Testing

We downloaded an open-source non-commercial version of the Studio 3T for MongoDB. The Studio 3T, one of the most popular MongoDB Graphical User Interface (GUI) tools, provides a visual editor to write and edit queries. This tool is used to connect to the same localhost of MongoDB, where our GENE2D database resides. The same query is implemented in our GENE2D system, and the Studio 3T and the results are compared. The aim is to check the functionality and accuracy of our query.

1. Query 1: Date type fields (display conditions, consanguinity, and inheritance pattern for patient date of birth before 2012 and Sort the results in descending order by DoB).

**Figure 11:**
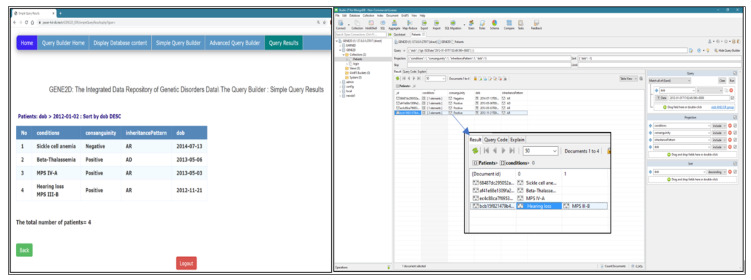
Testing query of date type field.

Testing the query in both systems delivered the same results for both the query and the sort order. The Studio 3T displays the number of the array elements, but to view the values, we have to double click on the condition elements to display it separately, as shown in Figure 11.

2. Query 2: array type fields (display conditions, tests and results for patients with a phenotype of “microcephaly” sort the results in ascending order by mother age).

**Figure 12:**
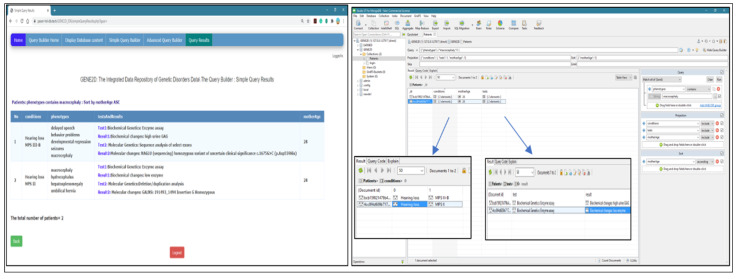
Testing query of the array type fields.

Results and field arrangement are the same in both systems. But as previously mentioned, Studio 3T rendering requires additional pages to display the exact contents of each array, as shown in Figure 12.

3. Query 3: advanced query with the logical operator (OR) (display conditions and phenotypes for patients with inheritance patterns “X_linked” OR who has “positive” consanguinity; and sort the results in descending by DoB).

**Figure 13:**
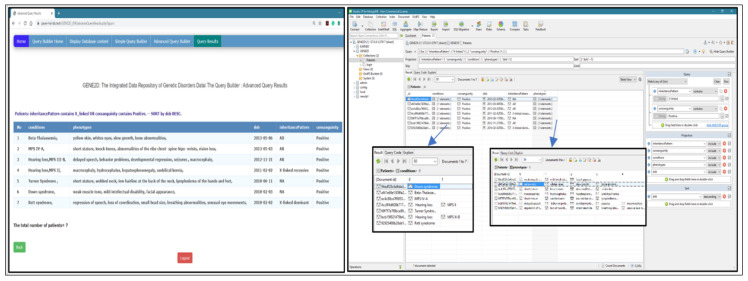
Testing advanced query with the logical operator (OR).

The results include all patients with “X_linked” inheritance patterns plus all patients with “positive” consanguinity. Both systems delivered the same results in the same order as presented in Figure 13.

4 Query 4: advanced query field type (array of objects) with a logical operator (display conditions for male patients who have undergone gene analysis tests; and sort the result in descending order by DoB).

**Figure 14:**
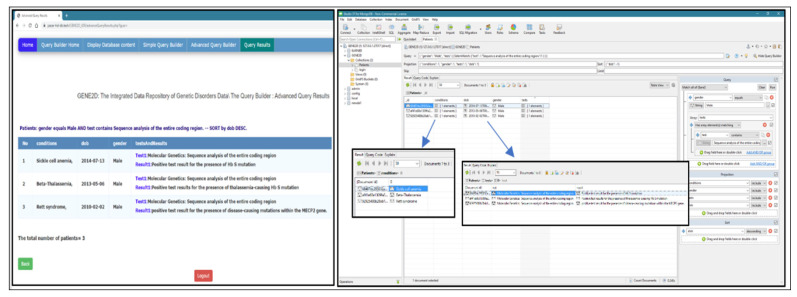
Testing advanced query, field type array of objects with a logical operator.

The results are displayed in our GENE2D system in a table format, male patients who had undergone “gene analysis” sorted by dob DESC. Studio 3T gives the same results but to show the array of objects (tests and their results), we have to go through multiple layers of pages, as shown in Figure 14.

##### Interface Testing

*Input Validation*

The selection from the menus limits user errors while entering the information required for the query. The validation of data entry, especially input text boxes, is crucial, as a small entry mistake may result in a fatal error, particularly when dealing with databases such as MongoDB. Passing special characters such as (\ */| ~) may result in an unrecognized error. Therefore, we increase the data selection option and minimize data entry.

*Alert Messages*

**Figure 15:**
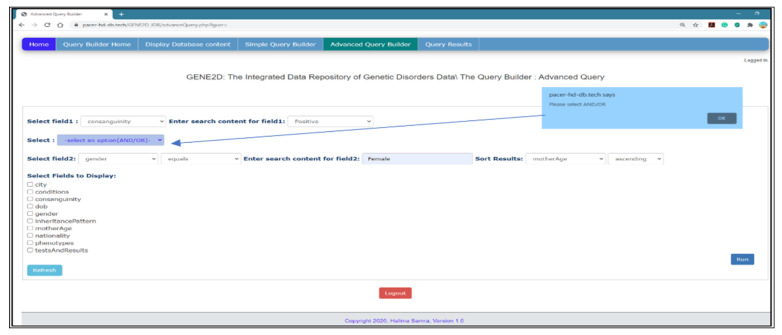
Warning messages.

*Warning messages*: alert messages are used to draw the user’s attention to certain erroneous actions which the user has performed and informs them of what to do, for example, if the user forgets to select a field option and presses the “Run” button or on the Advanced Query Builder page if the user selects two fields and forgets to select the logical operator. An example is presented in Figure 15.

**Figure 16:**
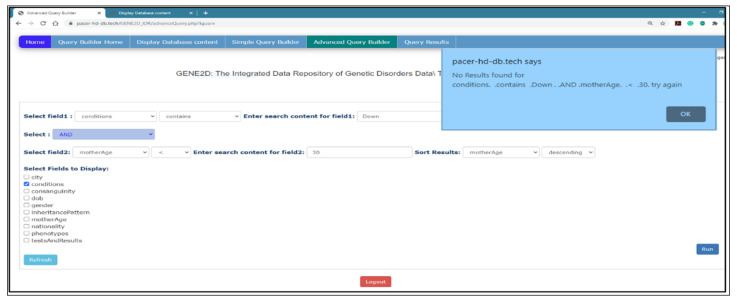
Informing messages.

*Informing messages*: when the user submits a query request to proceed, but there are no results found for that specific question in the database, an interactive message pops up, stating the user selection, but no results are found for that selection. An example is displayed in Figure 16.

#### 7.3.3. System Deployment

##### The GENE2D System Deployment on Virtual Private Server (VPS)

Our first step in deploying the GENE2D system is to consider several aspects such as using multiple resources for managing the MySQL server, the MongoDB server, and the Web server, but all these requirements cannot be fulfilled in a shared server where all the resources are limited to what the host provides. Therefore, we implemented the system on the virtual private server (VPS) to ensure system connectivity because all the resources required to run the GENE2D system are located in the same physical server.

##### The GENE2D System Deployment on the Cloud

As the GENE2D system grows and its website traffic surges and requires more storage space than VPS capacity, the service must move to the cloud environment. The cloud servers use several servers in a cluster to offer unlimited storage and maximum bandwidth and to manage load balancing. So, with cloud hosting, the GENE2D system expansion will not encounter any issues due to the cloud features allocating the load to any number of machines as the system needs to balance the flow and handle the traffic, as well as provide scalability with unrestricted storage capacity, and increased reliability, performance. To this end, the GENE2D system, which is a MongoDB-based deployment on the cloud, can be managed using MongoDB Atlas with a fully automated on-demand through a pay-as-you-go model cloud service. The MongoDB Atlas server allows the deployment of the GENE2D system on cloud platforms such as Amazon Web Services (AWS), Google Cloud Platform (GCP), and Microsoft Azure. Also, MongoDB Atlas offers built-in security controls and intuitive tools to work with data and extend and update the GENE2D application. It also offers visualization tools with MongoDB Charts, reliability with distributed fault tolerance and automated data recovery, performance based on, scale on-demand and elastic scalability, and efficiency through the automated deployments and database management services [37]. Based on these features, the migration of the GENE2D system from the VPS to the cloud, whenever needed, is a straightforward task with the MongoDB Atlas.

## 8. Conclusions

Knowing that data are available in one centralized location helps facilitate the research process for Saudi investigators in the area of genetic diseases. Our contribution in this regard is to propose a NoSQL-based integrated data repository of genetic disorders data, the GENE2D system. The purpose of the system is to integrate data from multiple local sources and provide a unified view of large data sets of genetic diagnostic data for Saudi patients with genetic disorders. Providing comprehensive datasets for data on genetic disorders in one place will assist in advancing research studies and develop and evaluate methods for diagnosis depending on patients’ clinical characteristics or phenotypes, diagnostics tests and results, and family history. The GENE2D system aims to provide an easy-to-use visual query interface based on the NoSQL document database, MongoDB. Our design methodology consists of three main steps, namely the process of extracting, transforming and loading data. First, data is acquired from multiple G3DMSs. Then a physical schema is modelled by embedding techniques before mapping and loading the data to the GENE2D system. Next, in the implementation and testing steps, the actual system is created and tested using validation tests to examine the performance of the query and verify the system interface interaction with expected user actions. Finally, the GENE2D system is currently deployed on a private virtual server (PVS), and as the system grows, the option of cloud deployment is managed using MongoDB Atlas to accommodate any future changes and growing requirements such as increased scalability, availability, security, and performance efficiency. Henceforth, the GENE2D system can receive data from new sources due to its flexible schema and indexing capabilities. The ultimate objective for the GENE2D system is to grow and develop the national genetic disorders database in Saudi Arabia.

## Figures and Tables

**Table 1:** List of fields extracted from the G3DMS (genetic disorders diagnosis data management system).

Field Name	Type	Relationship
Masked (MRN) → _id	primary key	patient’s proprietary
gender	Attribute	Patient’s proprietary
DoB	Attribute	Patient’s proprietary
city	Attribute	Patient’s proprietary
nationality	Attribute	Patient’s proprietary
consanguinity	Attribute	Patient’s proprietary
inheritancePattern	Attribute	Patient’s proprietary
motherAge	Attribute	Patient’s proprietary
phenotypes [pheno1, pheno2, …]	Entity	One-to-Many
conditions [cond1, cond2, …]	Entity	One-to-Many
tests [{test 1, result 1}, {test 2, result 2}, …]	Entity	One-to-Many

MRN: medical record number. DoB: date of birth. Transferred data fields from G3DMS tables [15].
